# The Modified Abbreviated Math Anxiety Scale: A Valid and Reliable Instrument for Use with Children

**DOI:** 10.3389/fpsyg.2017.00011

**Published:** 2017-01-19

**Authors:** Emma Carey, Francesca Hill, Amy Devine, Dénes Szűcs

**Affiliations:** Department of Psychology, Centre for Neuroscience in Education, University of CambridgeCambridge, UK

**Keywords:** math anxiety, AMAS, mAMAS, factor analysis, educational psychology, mathematics, anxiety

## Abstract

Mathematics anxiety (MA) can be observed in children from primary school age into the teenage years and adulthood, but many MA rating scales are only suitable for use with adults or older adolescents. We have adapted one such rating scale, the Abbreviated Math Anxiety Scale (AMAS), to be used with British children aged 8–13. In this study, we assess the scale's reliability, factor structure, and divergent validity. The modified AMAS (mAMAS) was administered to a very large (*n* = 1746) cohort of British children and adolescents. This large sample size meant that as well as conducting confirmatory factor analysis on the scale itself, we were also able to split the sample to conduct exploratory and confirmatory factor analysis of items from the mAMAS alongside items from child test anxiety and general anxiety rating scales. Factor analysis of the mAMAS confirmed that it has the same underlying factor structure as the original AMAS, with subscales measuring anxiety about Learning and Evaluation in math. Furthermore, both exploratory and confirmatory factor analysis of the mAMAS alongside scales measuring test anxiety and general anxiety showed that mAMAS items cluster onto one factor (perceived to represent MA). The mAMAS provides a valid and reliable scale for measuring MA in children and adolescents, from a younger age than is possible with the original AMAS. Results from this study also suggest that MA is truly a unique construct, separate from both test anxiety and general anxiety, even in childhood.

## Introduction

Math is an important skill not only for academic success, but also for efficient functioning in everyday life. Yet, a significant proportion of the population experience fear and apprehension when faced with numerical problems (Hembree, 1990; Ashcraft, 2002). This adverse emotional reaction toward math is more formally known as “math anxiety” (MA) and has been found to interfere with math performance as well as leading individuals to avoid math altogether (Hembree, 1990; Ashcraft, 2002).

### Math anxiety, test anxiety, and general anxiety

MA is by definition distinct from other forms of anxiety, since it is defined in terms of an emotional response elicited *by math* in particular. However, other forms of anxiety are in practice often associated with MA. For example, test anxiety relates to apprehension in evaluative settings (Putwain and Daniels, 2010; Brown et al., 2011). Studies have found a moderate positive correlation between MA and test anxiety (Hembree, 1990; Kazelskis et al., 2000; Devine et al., 2012). Research from Kazelskis et al. (2000) calls into question whether MA and test anxiety are truly distinct constructs; these researchers found that correlations between MA and test anxiety were almost as high as those within the MA measures themselves. Thus, for any measure of MA, it is important to test whether it is empirically dissociable from a measure of test anxiety: if impossible, this may reflect that MA measures simply compute a specific form of test anxiety. For example, Stöber and Pekrun (2004) suggest that test anxiety “may be ‘hidden’ under names related to more specific forms of test anxiety—(such as) math anxiety.”

On the other hand, general anxiety is a much less specific type of anxiety and refers to an individual's disposition toward anxiety about events, behaviors, and competence (Spence, 1997). General anxiety is also related to MA, although correlations tend to be smaller than those between test anxiety and MA (Hembree, 1990). Genetic research shows that genetic and environmental factors associated with general anxiety also act to influence MA levels (Wang et al., 2014).

The definitional uniqueness of MA may seem at odds with its consistent empirical association with test and general anxiety. However, it is important to bear in mind that these associations are small to moderate and account for only some individual variability in MA level. For example, Hembree (1990) reports an *r*^2^-value of 0.37 between MA and test anxiety. This means that 37% of variation in MA can be explained by variation in test anxiety scores: in other words, 63% of the variability in individuals' levels of MA comes from other sources. These sources have been highly debated in the math anxiety literature and go beyond the scope of this paper (for review see Maloney and Beilock, 2012; Carey et al., 2016). In light of the relationship between test anxiety, general anxiety and MA, we believe that it is vitally important to quantify the relationship between an MA measurement instrument and measurement instruments for general and test anxiety.

### Measuring math anxiety in children

Several anxiety measures have been developed for use with children; however, many of these measures are excessively age-restricted or adequate statistics supporting their validity are not provided. For example, Ramirez et al. (2013) use an eight-item questionnaire developed for young children. However, the questions refer to anxiety elicited by specific math problems, e.g., “How would you feel if you were given this problem? *There are 13 ducks in the water. There are 6 ducks in the grass. How many ducks are there in all?*” This measurement instrument is clearly only applicable to very young children—older children, regardless of their anxiety levels, are likely to be put at ease by the simplicity of the example problem. Furthermore, the authors reported reliability statistics only, leaving the validity of the measure in question.

The same problem regarding age specificity applies to the Scale for Early Mathematics Anxiety (MA; Wu et al., 2012), which asks students to rate how anxious they feel when asked to perform specific tasks (e.g., cutting an apple pie into four equal slices) or answer specific questions (e.g., “Is this right? 9 + 7 = 18.”). These authors do provide a factor analysis of the measure, but its age restriction to second and third grade children means it can never be used to look at MA in a sample with a wider age range. In particular, the limitations of these questionnaires mean it is impossible to gauge how MA changes with age.

An alternative measure of MA in children is the Math Anxiety Questionnaire (Thomas and Dowker, 2000). However, there is a distinct lack of psychometric research on the English version of this questionnaire (Wu et al., 2012). Furthermore, unlike other measures of MA, this questionnaire tends not to show any relationship between MA and math performance (Thomas and Dowker, 2000). This places the questionnaire's construct validity into question, since the relationship between MA and performance is long established in adolescents and adults (see Hembree, 1988; Carey et al., 2016 for review) and has also been observed in children when other questionnaires are used (Wu et al., 2012; Ramirez et al., 2013).

### Development of the mAMAS

Several measures of MA have been used in adult research, including the Abbreviated Math Anxiety Scale (AMAS; Hopko et al., 2003). The AMAS's short length makes it ideal (the value of short scales, alongside some common pitfalls, is discussed in Widaman et al., 2011). The AMAS was originally developed to have a two factor structure, using the highest loading items from the MA Rating Scale (MARS; Richardson and Suinn, 1972). When an exploratory factor analysis was run on the AMAS, a two-factor solution was found to be optimal, which was interpreted in terms of the two factors on which the scale development was based, learning MA (Learning subscale), and math evaluation anxiety (Evaluation subscale; Hopko et al., 2003). Since its development, the AMAS has been translated into several languages for use with different populations. Polish, Italian, and Persian translations of the AMAS have been found to be valid and reliable (Vahedi and Farrokhi, 2011; Primi et al., 2014; Cipora et al., 2015).

The modified AMAS (mAMAS) was developed in response to the need for a brief and appropriate scale to assess MA in British children and adolescents. Adjustments were made to the content of the AMAS in order to make the language appropriate to children speaking British English. Furthermore, the language and content of the scale has been adapted such that it is applicable across a broader age range (from middle childhood across adolescence), by altering references to specific topics in math (e.g., equations and algebra) and altering an item which refers to using tables in the back of a textbook, something which primary school aged British children have not encountered. Table 1 shows each item in the AMAS and mAMAS. We have previously used the mAMAS for British 8–11 year olds (Zirk-Sadowski et al., 2014), but its factor structure has not been investigated.

**Table 1:** **Items in the original and modified AMAS**.

**Item**	**Original AMAS**	**Modified AMAS**
1	Having to use the tables in the back of a math book	Having to complete a worksheet by yourself
2	Thinking about an upcoming math test 1 day before^*^	Thinking about a maths test the day before you take it
3	Watching the teacher work an algebraic equation on the blackboard	Watching the teacher work out a maths problem on the board
4	Taking an examination in a math course^*^	Taking a maths test
5	Being given a homework assignment of many difficult problems that is due the next class meeting^*^	Being given maths homework with lots of difficult questions that you have to hand in the next day
6	Listening to a lecture in math class	Listening to the teacher talk for a long time in maths
7	Listening to another student explain a math formula	Listening to another child in your class explain a maths problem
8	Being given a “pop” quiz in math class^*^	Finding out that you are going to have a surprise maths quiz when you start your maths lesson
9	Starting a new chapter in a math book	Starting a new topic in maths

* *Items measuring math evaluation anxiety (Evaluation subscale). All items not marked with asterisk measure math learning anxiety (Learning subscale)*.

### The current study

To evaluate construct validity of the mAMAS, we conduct confirmatory factor analysis to show for the first time that the mAMAS used with children and adolescents has the same factor structure as the AMAS used with adults. Furthermore, our unusually large sample size enabled us to divide the sample to conduct both exploratory and confirmatory factor analysis on items from the mAMAS alongside items from two other anxiety scales—the Child Test Anxiety Scale (CTAS; Wren and Benson, 2004) and the shortened form of the Revised Children's Manifest Anxiety Scale—Second Edition (RCMAS-II; Reynolds and Richmond, 2012). In doing this, we show that the mAMAS loads on to a unique factor—MA—dissociable from those factors measured by test and general anxiety scales. Our unique exploration of mAMAS's convergent and divergent validity provides strong evidence of the mAMAS's utility in measuring MA in childhood and adolescence.

## Methods

### Sample

We tested 1849 students in schools across Cambridgeshire (eight schools), Hertfordshire (seven schools), Suffolk (seven schools), Norfolk (two schools), and Bedfordshire (one school). Demographics of the schools varied widely. Using the number of children receiving Free School Meals (FSM) as an indicator of socioeconomic status, schools in our sample ranged from 2.9 to 36.5% receiving FSM (Department for Education, 2015b) compared with the national average of 20.9% (calculated from figures in Department for Education, 2015a). There was also a wide variation in schools' percentages of students with special educational needs (SEN) and English as an additional language (EAL). Students were only excluded on the basis of SEN or EAL if they were unable to understand or complete the tasks. Ethical permission was obtained from the Psychology Research Ethics Committee of the University of Cambridge, and opt-out consent was used.

Our sample consisted of students from two different age groups. The first of these (aged 8–9 years) consisted of students in year 4 of primary school. This group was chosen because they are old enough to complete standardized tests and questionnaires but are still in the early stages of education, therefore enabling us to capture MA in fairly young children. The second age group (age 11–13) consisted of students in years 7 and 8 of secondary school. This group was chosen in order to investigate how students' MA has developed by early secondary school.

Dealing with missing data appropriately and splitting of the sample for some analyses resulted in different sized samples for each analysis. Assessments of the reliability and factor structure of the mAMAS have a sample size of 1746 after casewise deletion of those with missing relevant data. Of the 824 primary school (year 4) students, there were 419 boys and 405 girls, with a mean age of 109.4 months (*SD* = 3.7 months). Of the 922 secondary school (year 7 and 8) students there were 463 boys and 459 girls, with a mean age of 148.1 months (*SD* = 4.0 months).

Analysis of the divergent validity of the mAMAS relied on item-level data from the mAMAS, RCMAS, and CTAS. The sample size after casewise deletion of those with missing items on any of these measures was 1469. The sample was stratified by school and then divided randomly to form two subsamples. The first of these was used for the exploratory factor analysis. This sample consisted of 735 students, 365 of whom were male, and 370 female. Three hundred and fifty-seven students were in year 4 and 378 in year 7 or 8. The mean age of this sample was 129.4 months (*SD* = 19.7 months). The second subsample, used for confirmatory factor analysis, consisted of the remaining 734 students, 369 of whom were male and 365 female. Three hundred and fifty-seven students were in year 4 and 377 in year 7 or 8. The mean age of this sample was 129.3 months (*SD* = 19.7 months).

### Materials

#### Math anxiety

MA was measured using a modified version of the Abbreviated Math Anxiety Scale (AMAS; Hopko et al., 2003); a self-report questionnaire with a total of nine items. Participants use a 5-point Likert scale to indicate how anxious they would feel during certain situations involving math (1 = low anxiety to 5 = high anxiety). Research indicates that the original AMAS is as effective as the longer Math Anxiety Rating Scale (MARS; Hopko et al., 2003 e.g., internal consistency: Cronbach α = 0.90; 2 week test-retest reliability: *r* = 0.85; convergent validity of AMAS and MARS-R: *r* = 0.85).

#### Test anxiety

Test anxiety was measured using the Children's Test Anxiety Scale (CTAS; Wren and Benson, 2004). This 30-item self-report questionnaire assesses children's thoughts (e.g., “When I take tests I worry about doing something wrong”), autonomic reactions (e.g., “When I take tests my belly feels funny”), and off-task behaviors (e.g., “When I take tests I tap my feet”) in various testing situations. Participants respond to items using a four-point scale (almost never—almost always). Adequate reliability and internal construct validity has been confirmed using both “development” and “validation” samples (Wren and Benson, 2004; Cronbach α = 0.92).

#### General anxiety

General anxiety was measured using the Short Form of the Revised Children's Manifest Anxiety Scale: Second Edition (RCMAS-2; Reynolds and Richmond, 2012); a self-report 10-item measure. The Short Form was used in place of the full 49-item questionnaire in order to minimize testing time. Participants respond using a simple yes/no response format. Adequate reliability has been demonstrated for the Short Form (Reynolds and Richmond, 2012; Cronbach α = 0.82).

### Procedure

Researchers went to schools to administer the testing in group settings (either as a class or whole year group). As well as completing the questionnaires analyzed here, students also completed the age-appropriate Hodder Group Reading Test (Vincent and Crumpler, 2007) and Mathematics Assessment for Learning and Teaching (Williams et al., 2005). Testing sessions took ~2 h and the order of tests and questionnaires was counterbalanced between schools. We made sure to present material in an age-appropriate manner for both age groups of children. For the year 4 students, this included giving a colorful PowerPoint slide-show about the tasks, giving practice questionnaire items and reading all questionnaire items aloud. For both age groups, any difficult words or terms (e.g., “anxiety”) were defined and explained in terms of more common words for the age groups involved (i.e., feelings of worry, fear, or nerves). The questionnaires were presented in a readable questionnaire booklet. We also included emoticons on the mAMAS and CTAS Likert-scales to remind students of which end of the scale reflected positive emotions and which end reflected negative emotions (see Supplementary Image 1 for a copy of the mAMAS as presented to students; the same emoticons as can be seen on this scale were also used on the CTAS). Furthermore, students were always told that they could ask researchers if they had any questions or could not read/understand any items.

### Analysis

#### Reliability of the mAMAS

The reliability of the mAMAS was assessed using both ordinal alpha and Cronbach alpha (as in Cipora et al., 2015). The rationale for using ordinal alpha as well as Cronbach alpha is that the latter relies on Pearson's correlation coefficients between items, thus assuming continuous data. The mAMAS measures items on a Likert-type scale, violating this assumption. Cronbach alpha is also shown to be reduced both by scales with few items (Yang and Green, 2011) and where data is not normally distributed (Sheng and Sheng, 2012). For these reasons, we also prioritized ordinal alpha for the scale (Gadermann et al., 2012).

#### Construct validity of the mAMAS

As well as making an assessment of the reliability of the mAMAS, we investigated its validity by carrying out a confirmatory factor analysis based on the two-factor structure of the original AMAS (Hopko et al., 2003). The factor structure of the original AMAS questionnaire involved correlated latent variables representing Learning and Evaluation. See Table 1 for details of each item and its associated subscale.

Mplus was used to conduct this analysis, employing theta parameterization and weighted least squares means and variance adjusted (WLSMV) estimation due to the categorical nature of Likert-scale variables (Muthén and Asparouhov, 2002; Muthén and Muthén, 2015). WLSMV estimation performs well in structural equation modeling of ordinal variables, even in the case of complex models with small sample sizes (Nussbeck et al., 2006). Age (year 4 vs. year 7 and 8) was used as a grouping variable in the analysis.

#### Divergent validity of the mAMAS: exploratory factor analysis

R was used to conduct all analyses (R Core Team, 2016). Item-level variables from the RCMAS were binary and those from the CTAS and mAMAS polytomous. Thus, we used the R package polycor (Fox, 2010) to create a matrix of tetrachoric and polychoric correlations between the 49 variables (10 from the RCMAS, 30 from the CTAS and 9 from the mAMAS). Because our goal was to examine the latent variables underlying questionnaire results, rather than simple data reduction, factor analysis was deemed preferable to principal components analysis (Costello and Osborne, 2005; Osborne, 2014). R's psych package (Revelle, 2015) was used to conduct this exploratory factor analysis.

Principal axis factoring was used; this was favored over maximum likelihood extraction, because of its insensitivity to violations of the assumption of multivariate normality (Osborne, 2014). Promax rotation was used, since evidence suggests that this oblique rotation method performs preferably to the more common varimax rotation in identifying a simple structure, particularly when factors have a correlation above 0.3 (Swygert et al., 2001; DeVellis, 2003; Finch, 2006; Tabachnick and Fidell, 2007). Prior research shows a correlation between general anxiety, test anxiety and MA (Hembree, 1990; Kazelskis et al., 2000) as well as between items and subscales of the CTAS and AMAS (Hopko et al., 2003; Wren and Benson, 2004), leading us to the expectation that factors underlying responses to the RCMAS, CTAS, and mAMAS are likely to be correlated.

Two methods were used to determine the optimal number of factors to extract. We chose to use Horn's parallel analysis (Horn, 1965) and Velicer's Minimum Average Partial (MAP) test (Velicer, 1976) using the nFactors and psych packages of R (Raiche, 2010; Revelle, 2015). These are regarded as two of the best techniques to determine the number of factors underlying a dataset (Zwick and Velicer, 1986; Osborne, 2014). Horn's Parallel Analysis and Velicer's MAP test are generally effective when the correlation matrix consists of polychoric correlations from ordinal data (Garrido et al., 2011, 2012).

#### Divergent validity of the mAMAS: confirmatory factor analysis

We conducted confirmatory factor analysis to yield a model for the mAMAS, CTAS, and RCMAS with the number of factors which had emerged from the exploratory factor analysis. Mplus was used for this analysis (Muthén and Muthén, 2015). As in the confirmatory factor analysis of the mAMAS alone, we used theta parameterization and the WLSMV estimator, as observed variables were binary or polytomous. Each item was allowed to correlate with a factor if, and only if, it had a factor loading >0.20 on that factor in the exploratory factor analysis. We saw no reason for items to be limited to loading on only one factor.

## Results

### Descriptive statistics for the mAMAS

**Figure 1:**
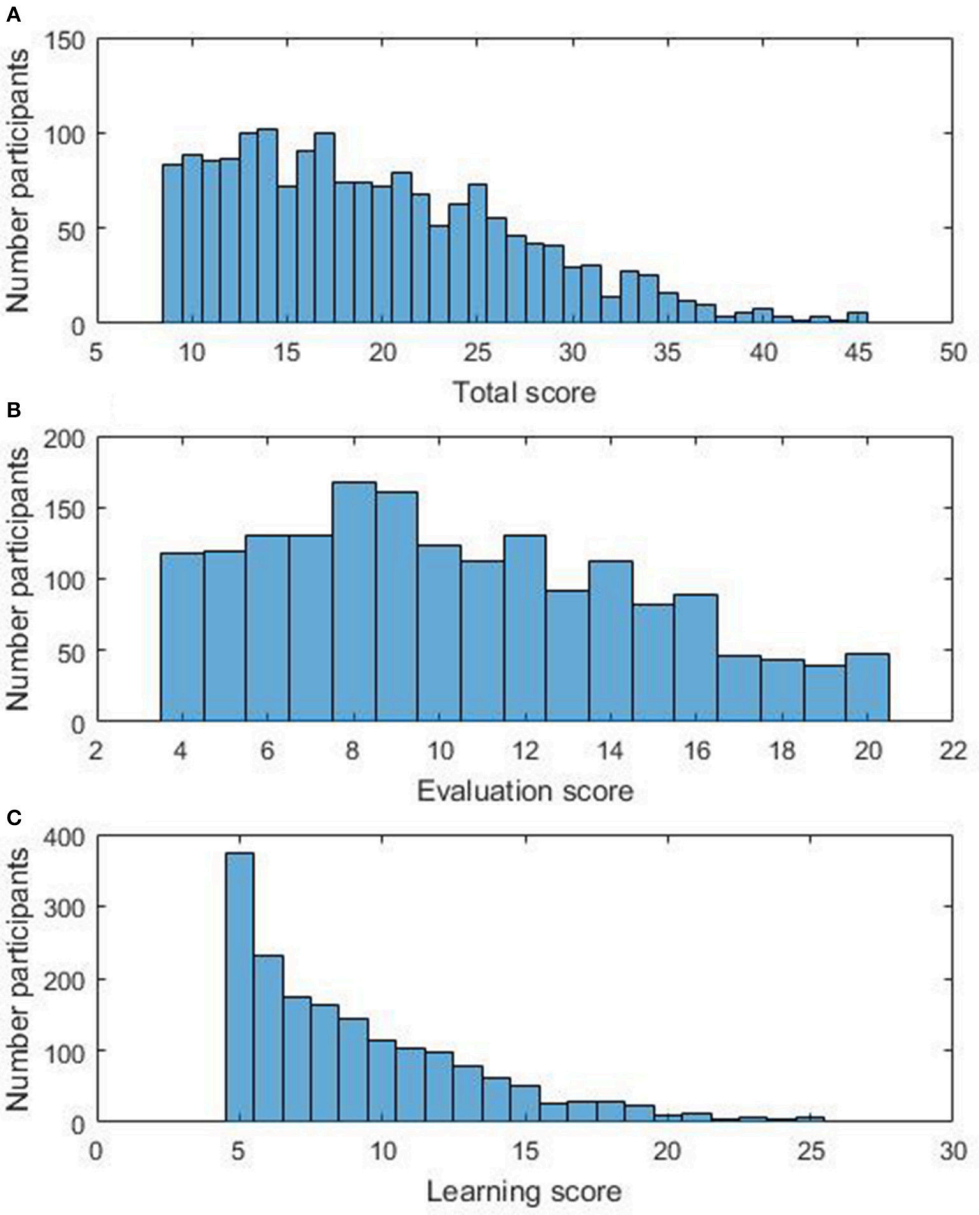
**Distribution of (A)** mAMAS Total scores, **(B)** mAMAS Evaluation scores, and **(C)** mAMAS Learning scores.

The average mAMAS total score was 19.67 (*SD* = 7.65). The average score for the Evaluation subscale was 10.48 (*SD* = 4.32) and for the Learning subscale was 9.19 (*SD* = 4.17). A Shapiro-Wilk test showed that mAMAS scores were not normally distributed, neither on the Total scale [*W*_(1746)_ = 0.95, *p* < 0.001] nor the Evaluation [*W*_(1746)_ = 0.96, *p* < 0.001] and Learning [*W*_(1746)_ = 0.87, *p* < 0.001] subscales. Distributions of Total and subscale scores can be seen in Figure 1.

With a Bonferroni corrected significance level of 0.008 for six comparisons (0.05/6 = 0.008; all *p*-values are uncorrected), mAMAS Total scores were not significantly different in year 7 and 8 (*M* = 20.02) than in year 4 [*M* = 19.26; *t*_(1744)_ = −2.07, *p* = 0.04]. mAMAS Evaluation scores were significantly higher in year 7 and 8 (*M* = 10.76) than in year 4 [*M* = 10.15; *t*_(1744)_ = −2.95, *p* = 0.003]. mAMAS Learning scores were not significantly different between year 7 and 8 (*M* = 9.11) and year 4 [*M* = 9.26; *t*_(1744)_ = −0.75, *p* = 0.45]. mAMAS Total scores were significantly higher in girls (*M* = 21.0) than boys [*M* = 18.36; *t*_(1744)_ = 7.29, *p* < 0.001]. mAMAS Evaluation scores were significantly higher in girls (*M* = 11.43) than boys [*M* = 9.55; *t*_(1744)_ = 9.33, *p* < 0.001]. mAMAS Learning scores were also significantly higher in girls (*M* = 9.57) than boys [*M* = 8.82; *t*_(1744)_ = 3.78, *p* < 0.001]. Total and subscale scores split by gender and age group can be seen in Table 2.

**Table 2:** **Descriptive statistics for the mAMAS, split by school year, and gender**.

			**Total**		**Evaluation**		**Learning**	
		***n***	**Average**	***SD***	**Average**	***SD***	**Average**	***SD***
Year 4	Female	404	20.70	7.49	11.14	4.17	9.56	4.08
	Male	420	17.88	7.87	9.21	4.29	8.67	4.26
Year 7 and 8	Female	459	21.25	7.69	11.68	4.37	9.57	4.29
	Male	463	18.80	7.11	9.85	3.98	8.95	3.98

### Reliability of the mAMAS

#### Ordinal alpha

First we examined ordinal alpha for the entire sample. Ordinal alpha for the total scale was 0.89, for the Learning subscale was 0.83 and for the Evaluation subscale was 0.83. Ordinal alpha was not increased by removing any item from either subscale or the total scale.

We then looked at ordinal alpha for each age group separately. In year 4 students, ordinal alpha for the total scale was 0.89, for the Learning subscale was 0.81, and for the Evaluation subscale was 0.83. In year 7/8 students, ordinal alpha for the total scale was 0.89, for the Learning subscale was 0.85 and for the Evaluation subscale was 0.84. Ordinal alpha-values were not increased by removing any item from either subscale or the total scale in either age group.

#### Cronbach alpha

Cronbach alpha for the whole scale was 0.85 (95% confidence interval 0.83–0.87), for the Learning subscale was 0.77 (95% confidence interval 0.74–0.80) and for the Evaluation subscale was 0.79 (95% confidence interval 0.76–0.83). Cronbach alpha was not increased by removing any item from either subscale or the total scale.

For year 4 students, Cronbach alpha for the total scale was 0.85 (95% confidence interval 0.82–0.87), for the Learning subscale was 0.74 (95% confidence interval 0.69–0.79) and for the Evaluation subscale was 0.78 (95% confidence interval 0.73–0.83). For year 7/8 students, Cronbach alpha for the total scale was 0.86 (95% confidence interval 0.83–0.88), for the Learning subscale was 0.80 (95% confidence interval 0.76–0.84) and for the Evaluation subscale was 0.81 (95% confidence interval 0.76–0.85). Cronbach alpha-values were not increased by removing any item from either subscale or the total scale in either age group.

### Factor structure of the mAMAS

**Figure 2:**
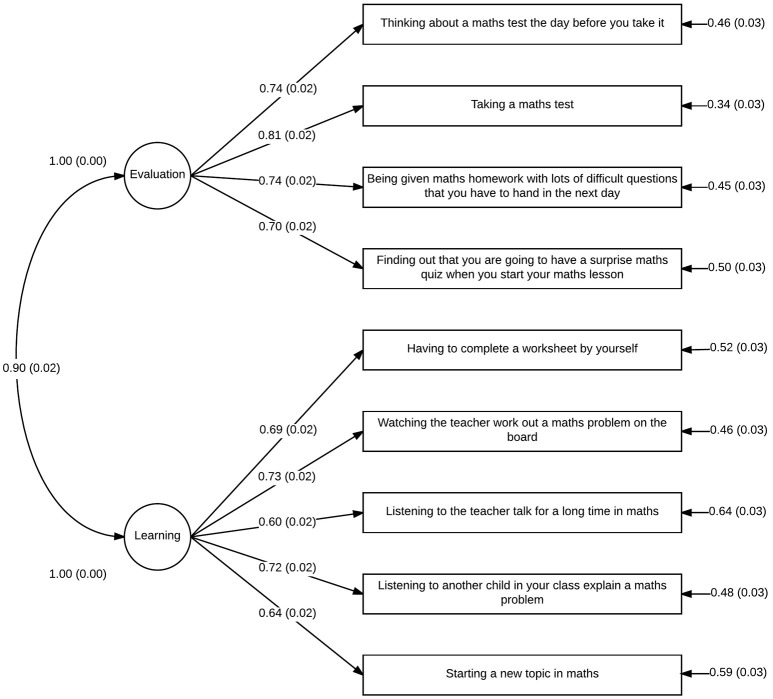
**Confirmatory factor analysis of mAMAS: path diagram for year 4 students**.

**Figure 3:**
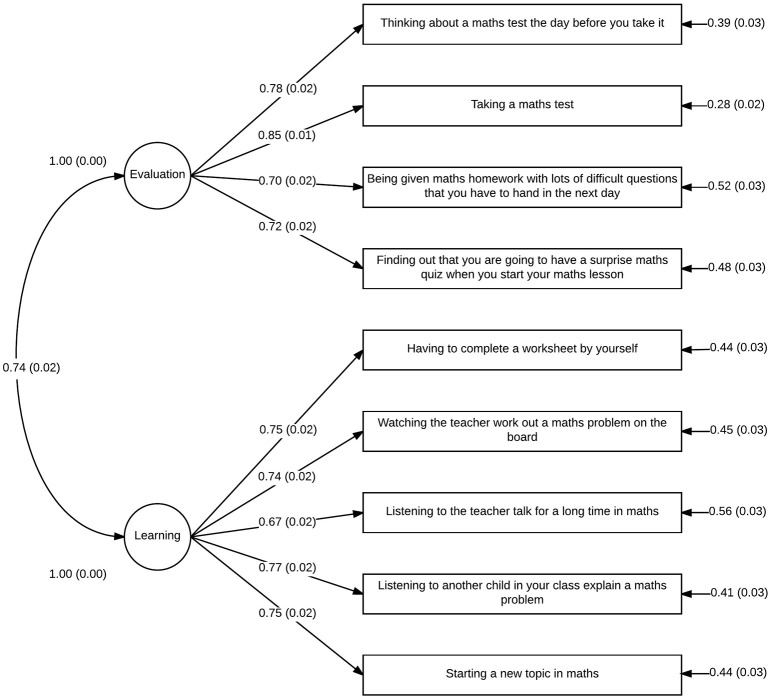
**Confirmatory factor analysis of mAMAS: path diagram for year 7/8 students**.

Figures 2, 3 show the model and standardized path coefficients for each observed variable for year 4 and year 7/8 students, respectively. All item loadings are at an acceptable level (≥0.60) and all parameter estimates were found to be significantly different from 0. As expected with such a large sample size (Bentler and Bonett, 1980), a χ^2^-test of model fit suggested that the model was significantly different from the ideal model: χ^2^ = 466.95(84, *N* = 1746), *p* < 0.001. However, root mean squared error of approximation (RMSEA) was 0.072 (90% confidence interval 0.066–0.079) and comparative fit index (CFI) was 0.97. These indices suggests acceptable model fit; although the RMSEA exceeds the more stringent model fit cut-off of 0.06 proposed by Hu and Bentler (1999), one should be careful when interpreting these suggestions as strict rules, rather taking them as guidelines which should not be overgeneralized (Marsh et al., 2004). SRMR is not reported for this analysis because Mplus is unable to calculate SRMR in analyses with a grouping variable.

### Reliability of the RCMAS and CTAS

Ordinal α for the RCMAS was 0.73 and Cronbach α was 0.74 (95% confidence interval 0.71–0.76), suggesting adequate reliability. Ordinal α for the CTAS was 0.92 and Cronbach α was 0.92 (95% confidence interval 0.91–0.93), suggesting excellent reliability.

### Exploratory factor analysis of the mAMAS, CTAS, and RCMAS

Horn's parallel analysis and Velicer's MAP test suggested that a 5-factor model would be optimal: thus we opted to examine the 5-factor model, in which all extracted factors had loadings >0.4 on 3 or more variables. Eigen-values of the five factors in this model ranged from 2.1 to 5.3. Items were easily clustered into factors.

The factors identified were perceived to represent: Test Anxiety, MA, Physical Anxiety, Off-Task Behaviors in Tests and Social Anxiety. These factors largely related to specific anxiety scales or subscales. The MA factor consisted of all mAMAS items and one item of the CTAS. Test Anxiety consisted largely of CTAS items and the two mAMAS items which addressed math tests. Items from the Autonomic Reactions subscale of the CTAS and the Physiological subscale of the RCMAS clustered onto the Physical Anxiety factor. Items from the Off Task Behaviors subscale of the CTAS formed the factor Off-Task Behaviors in Tests. Finally, items loading onto Social Anxiety were all from the RCMAS, with the highest loading items making reference to social situations. For a detailed view of each item's factor loadings, see Table 3.

**Table 3:** **Show factor loadings for each questionnaire item from exploratory factor analysis**.

**Test**	**Item**	**Test anxiety**	**Math anxiety**	**Physical anxiety**	**Off-task behaviors**	**Social anxiety**
mAMAS	Finding out that you are going to have a surprise maths quiz…		0.60			
mAMAS	Watching the teacher work out a maths problem on the board		0.70			
mAMAS	Starting a new topic in maths		0.65			
mAMAS	Having to complete a worksheet by yourself		0.61			
mAMAS	Being given maths homework with lots of difficult questions…		0.51			
mAMAS	Listening to another child in your class explain a maths problem		0.67			
mAMAS	Listening to the teacher talk for a long time in maths		0.55			
mAMAS	Thinking about a maths test the day before you take it	0.25	0.45			
mAMAS	Taking a maths test	0.40	0.45			
CTAS	It is hard for me to remember the right answers	0.25	0.28			
CTAS	I wonder if my answers are right	0.58				
CTAS	I think about what my grade will be	0.60				
CTAS	I worry about how hard the test is	0.55				
CTAS	I worry about doing something wrong	0.66				
CTAS	I think about what will happen if I fail	0.72				
CTAS	I worry about failing	0.85				
CTAS	I worry about what my parents will say	0.39				
CTAS	I wonder if I will pass	0.58				
CTAS	I think that I should have studied more	0.38				
CTAS	I think most of my answers are wrong	0.56				
RCMAS	I am nervous	0.27				
CTAS	I think I am going to get a bad grade	0.70				
CTAS	I think about how poorly I am doing	0.48				
CTAS	My heart beats fast	0.38			0.52	
CTAS	I feel nervous	0.59			0.31	
CTAS	I feel scared	0.48			0.46	
CTAS	My hand shakes				0.56	
CTAS	I feel warm				0.60	
CTAS	My face feels hot				0.56	
RCMAS	I have too many headaches				0.34	
CTAS	My belly feels funny				0.64	
RCMAS	Often I feel sick in my stomach				0.44	
CTAS	My head hurts				0.50	
RCMAS	I wake up scared sometimes				0.35	
CTAS	I find it hard to sit still			0.63		
CTAS	I tap my feet			0.56		
CTAS	I look around the room			0.68		
CTAS	I have to go to the bathroom			0.22		
CTAS	I try to finish up fast			0.41		
CTAS	I stare			0.56		
CTAS	I play with my pencil			0.72		
CTAS	I look at other people			0.57		
CTAS	I check the time	0.22		0.42		
RCMAS	I often worry about something bad happening to me	0.20				0.20
RCMAS	I feel someone will tell me I do things the wrong way					0.25
RCMAS	I get nervous around people					0.28
RCMAS	I worry that others do not like me					0.53
RCMAS	I fear other kids will laugh at me in class					0.83
RCMAS	I fear other people will laugh at me					0.95

*All loadings ≥0.20 are reported*.

### Confirmatory factor analysis of the mAMAS, CTAS, and RCMAS

Standardized root mean square residual (SRMR) for the model was 0.06, RMSEA was 0.04 (90% CI 0.039–0.043) and CFI 0.94. Whilst CFI was just below Hu and Bentler's (1999) cut-off of 0.95, the conjunction of SRMR and RMSEA at these low levels suggests that the model has adequate fit. Standardized path coefficients are reported in Table 4.

**Table 4:** **Show standardized path coefficients between factors and for each factor an item was specified to be related to in the confirmatory factor analysis of the RCMAS, CTAS, and mAMAS**.

**Test**	**Factor or item**	**Test anxiety**	**Math anxiety**	**Physical anxiety**	**Off-task behaviors**	**Social anxiety**
	Math anxiety	0.67				
	Physical anxiety	0.69	0.57			
	Off-task behaviors	0.49	0.57	0.51		
	Social anxiety	0.58	0.50	0.51	0.37	
mAMAS	Finding out that you are going to have a surprise maths quiz…		0.72			
mAMAS	Watching the teacher work out a maths problem on the board		0.65			
mAMAS	Starting a new topic in maths		0.66			
mAMAS	Having to complete a worksheet by yourself		0.73			
mAMAS	Being given maths homework with lots of difficult questions…		0.75			
mAMAS	Listening to another child in your class explain a maths problem		0.65			
mAMAS	Listening to the teacher talk for a long time in maths		0.56			
mAMAS	Thinking about a maths test the day before you take it	0.43	0.42			
mAMAS	Taking a maths test	0.55	0.26			
CTAS	It is hard for me to remember the right answers	0.32	0.30			
CTAS	I wonder if my answers are right	0.59				
CTAS	I think about what my grade will be	0.55				
CTAS	I worry about how hard the test is	0.75				
CTAS	I worry about doing something wrong	0.74				
CTAS	I think about what will happen if I fail	0.78				
CTAS	I worry about failing	0.83				
CTAS	I worry about what my parents will say	0.67				
CTAS	I wonder if I will pass	0.49				
CTAS	I think that I should have studied more	0.62				
CTAS	I think most of my answers are wrong	0.79				
RCMAS	I am nervous	0.62				
CTAS	I think I am going to get a bad grade	0.74				
CTAS	I think about how poorly I am doing	0.77				
CTAS	My heart beats fast	0.32		0.41		
CTAS	I feel nervous	0.51		0.28		
CTAS	I feel scared	0.41		0.49		
CTAS	My hand shakes			0.65		
CTAS	I feel warm			0.52		
CTAS	My face feels hot			0.68		
RCMAS	I have too many headaches			0.39		
CTAS	My belly feels funny			0.73		
RCMAS	Often I feel sick in my stomach			0.64		
CTAS	My head hurts			0.69		
RCMAS	I wake up scared sometimes			0.44		
CTAS	I find it hard to sit still				0.72	
CTAS	I tap my feet				0.56	
CTAS	I look around the room				0.59	
CTAS	I have to go to the bathroom				0.66	
CTAS	I try to finish up fast				0.58	
CTAS	I stare				0.72	
CTAS	I play with my pencil				0.51	
CTAS	I look at other people				0.56	
CTAS	I check the time	0.21			0.24	
RCMAS	I often worry about something bad happening to me	0.16				0.36
RCMAS	I feel someone will tell me I do things the wrong way					0.59
RCMAS	I get nervous around people					0.74
RCMAS	I worry that others do not like me					0.70
RCMAS	I fear other kids will laugh at me in class					0.88
RCMAS	I fear other people will laugh at me					0.93

## Discussion

### Reliability of the mAMAS

Ordinal alpha, the most appropriate measure for items on an interval scale, suggests that the internal consistency of the scale as a whole is very good (0.89) and that the subscales have good internal consistency (both 0.83). These high alpha-values suggest that, regardless of the factor structure of the scale, the mAMAS reliably measures one construct. This suggests that our modifications of the AMAS did not decrease the scale's internal consistency, and that the mAMAS is reliable even when children and adolescents are being tested. Furthermore, these alpha-values remained high when year 4 and year 7/8 students' results were analyzed separately. This suggests that the mAMAS is a reliable scale of MA both in middle childhood and early adolescence. This indicates that the mAMAS is preferable to other childhood MA scales such as the Child MA Questionnaire (Ramirez et al., 2013) and Scale for Early MA (Wu et al., 2012), which can only be used for around 2 academic years.

### Construct validity of the mAMAS

Our confirmatory factor analysis of the mAMAS based on the subscales identified in the original mAMAS and confirmed to exist in Polish, Iranian, and Italian translations of the AMAS (Hopko et al., 2003; Vahedi and Farrokhi, 2011; Primi et al., 2014; Cipora et al., 2015) suggest that the two-factor solution previously identified also applies to the mAMAS. Each item in the mAMAS loaded onto the same subscale as their counterparts in the original AMAS, with the two subscales representing Learning MA and mathematics Evaluation anxiety.

All factor loadings were at an acceptable level (≥0.60) which, alongside adequate measurements of model fit, suggests that the mAMAS can be conceptualized in terms of the same two subscales which comprise the original AMAS. This was the case for both younger (year 4) and older (year 7 and 8) children, suggesting that the mAMAS has good construct validity when used for children aged 8–13. This represents a very broad age range compared with other childhood MA scales, and highlights the utility of the mAMAS when researchers wish to investigate MA across development.

### Divergent validity of the mAMAS

In order to assess convergent and divergent validity of the mAMAS we analyzed children's scores on mAMAS items alongside items from the CTAS and the RCMAS-II short form. MA, test anxiety and general anxiety have previously been shown to be related, but should be dissociable. Thus, we had the expectation that if the mAMAS truly measures MA, mAMAS items should load onto one or more unique factors.

We first ran exploratory factor analysis on data from half of the sample, to explore how items were related without relying on prior theoretical assumptions. This was followed up with a confirmatory factor analysis (using the factors identified in exploratory factor analysis) on the other half of the sample. Adding a confirmatory factor analysis enabled us to confirm that the factor structure determined through exploratory factor analysis was not subject to overextraction of spurious factors and to gain measures of model fit.

The exploratory and confirmatory factor analyses of item-level data from the RCMAS, CTAS and mAMAS suggest that an individual's scores on each item of these questionnaires is influenced by multiple, unique but related factors. A 5-factor solution best explained the variance in the data without unnecessary complexity. These five factors were interpreted as representing: test anxiety, MA, off-task behaviors, physical anxiety, and social anxiety. This 5-factor solution was used to conduct confirmatory factor analysis, and it was determined that the model had a good fit to the data.

It is notable that all items in the mAMAS loaded relatively highly on the MA factor (all items had a factor loading >0.40 in the exploratory factor analysis, and all but one item had a factor loading >0.40 in the confirmatory factor analysis). This suggests that the mAMAS taps into a unique area of anxiety, even in children aged 8–13. If MA could be explained in terms of other anxiety forms, such as test anxiety and general anxiety, one would expect no unique MA factor to emerge from a factor analysis. Therefore, the analysis suggests that the mAMAS shows divergent validity: it measures a form of anxiety which can be differentiated from test and general anxiety.

Two items in the mAMAS had a similar loading on the Test Anxiety factor as they did on the MA factor. These items, “Thinking about a math test the day before you take it” and “Taking a math test,” make explicit references to both mathematics and evaluative situations. It is unsurprising that they load similarly onto MA and Test Anxiety factors, because being high in either MA *or* test anxiety would influence one's response to these items. This might suggest that the Evaluation subscale of the mAMAS is influenced by test anxiety as much as by MA, and that the Learning subscale provides a purer measure of MA.

Our findings that items from the mAMAS almost all loaded onto a unique factor representing MA provide strong empirical evidence for two things. Firstly, MA appears to exist as a unique anxiety form. Some items measured by MA questionnaires might measure two different forms of anxiety (MA and test anxiety), but other items measure MA alone, suggesting that MA can be considered as a separate construct to test anxiety. This calls into question how much of the relationship between MA and test anxiety would remain if questions which tap into both anxiety forms were removed from MA questionnaires. Secondly, the mAMAS taps into this unique MA factor, rather than merely reflecting another form of anxiety. Thus, we have shown both that MA exists in its own right in children and adolescents and that we are able to capture it using the mAMAS.

### Implications for psychiatry, educational psychology, and research

Having a valid and reliable scale with which to measure MA is of vital importance to researchers, psychiatrists, and educational psychologists. MA is associated with a variety of negative outcomes, including avoidance of math-related situations and poorer outcomes in math (Hembree, 1990). Childhood and adolescence is the optimal time to tackle MA, as children and teenagers are still in full-time education and enrolled in compulsory math classes. Having a valid and reliable short measurement instrument enables researchers, educational psychologists and educational practitioners to easily assess MA in the children they are working with, in order to develop and implement interventions.

This larger age span of the mAMAS compared with other child MA questionnaires could be very beneficial to both educational practitioners and researchers. In the school or educational psychology setting, having different measures for each age group is likely to cause confusion. It also raises questions around which measure is appropriate for a child who functions at a lower or higher academic level than their peers: is it more appropriate to administer a questionnaire suitable for their chronological age or their academic level? For example, a child with very strong mathematical ability may be anxious in response to a question they find challenging. If the sample questions in an anxiety questionnaire are those which would stretch the average child of their *age* rather than their ability, their answers may reflect a lack of anxiety simply because they find the questions easy. Having a questionnaire which does not refer to specific math problems is, therefore, ideal.

In addition, the AMAS is a very common tool for researchers of adult MA. The fact that the mAMAS is similar to the AMAS in both style, content and factor structure may enable researchers to better study how math anxiety changes from childhood to adulthood, by using two closely related scales.

### Limitations and further study

Assessing the test-retest reliability of the mAMAS would be useful, but practically challenging with a large sample such as used here. Taking another measure of MA could confirm the convergent validity of the mAMAS. However, as discussed, neither the Child MA Questionnaire (Ramirez et al., 2013) nor the Scale for Early MA (Wu et al., 2012) are appropriate for the age range of 8–13 years. The Mathematics Attitude and Anxiety Questionnaire (Thomas and Dowker, 2000) has not been validated in its English form. Although validations of German and Brazilian translations do exist, these do not span the full age range used in the current study (Wood et al., 2012). Therefore, whilst testing convergent validity against another scale would be optimal, the fact that no MA measure has been psychometrically tested for use from age 8 to 13 would make doing so practically impossible. As further childhood MA tests are developed, cross-validation will become possible.

Further studies of the mAMAS may wish to investigate more specific properties of the test, such as whether its factor structure is invariant across various groups of children. For example, average levels of MA have consistently been shown to be lower in boys than girls (see Hembree, 1990 for review) and it would be interesting to see whether the factor structure of MA questionnaires varies by gender or other grouping variables such as race, ethnicity, and socio-economic status. In addition, further studies conducting confirmatory factor analysis on the mAMAS, CTAS, and RCMAS based on our findings would be of great interest given the bias present in *k*-fold cross validation (Kohavi, 1995).

## Conclusions

Our analyses suggest that the mAMAS provides a valid and reliable measurement of MA in children aged 8–13. The mAMAS appears to have the same factor structure as the original AMAS. It also appears to tap uniquely into MA, forming a unique factor when items were factor analyzed alongside items from the CTAS and RCMAS. The questions in the mAMAS are phrased as broadly as possible and should be applicable to all English-speaking children and adolescents, as long as they are learning math in school and have the questions explained or read aloud to them when necessary. Thus, the mAMAS provides a useful assessment of MA, which may be utilized by researchers, educational psychologists, and educational practitioners.

## Ethics statement

Cambridge Psychology Research Ethics Committee.Children took an opt-out consent form home from school in their book bags, for their parents/guardians to return if they did not want their child to participate in the research. We worked with children whose parents/guardians did not opt-out of participation, as approved by Cambridge Psychology Research Ethics Committee.We made arrangements for any participant who appeared distressed or expressed that they did not wish to participate to leave with no penalty and return to other activities in school (testing carried out in the main school halls or classrooms). Students generally found the tasks (maths and reading tests and filling in questionnaires) within the realm of what they normally do in the school day.

## Author contributions

AD and DS made substantial contributions to the conception and design of the work. AD, FH, and EC made substantial contributions to the acquisition and interpretation of the data. EC and DS were involved in analysis of the data. EC drafted the work with contributions from FH. AD and DS were involved in critical revisions and discussion of intellectual content.

## Funding

This project has been funded by the Nuffield Foundation (EDU/41179), although the views expressed are those of the authors and not necessarily those of the Foundation. The project also received funding from the James S. McDonnel Foundation (220020370).

### Conflict of interest statement

The authors declare that the research was conducted in the absence of any commercial or financial relationships that could be construed as a potential conflict of interest.
