# Supplementary material for: The Modified Abbreviated Math Anxiety Scale: A Valid and Reliable Instrument for Use with Children
Source: Front Psychol. 2017 Jan 19;8:11. doi: 10.3389/fpsyg.2017.00011 (PMC5243819; doi:10.3389/fpsyg.2017.00011)
Supplement: Supplementary file 1 [file Image1.PDF]

Instructions:
Please give each sentence a score in terms of how anxious you would feel during each situation. Use the scale at the right side and circle the number which you think best describes how you feel.

	☺				☹
	Low anxiety	Some anxiety	Moderate anxiety	Quite a bit of anxiety	High anxiety
1. Having to complete a worksheet by yourself.	1	2	3	4	5
2. Thinking about a maths test the day before you take it.	1	2	3	4	5
3. Watching the teacher work out a maths problem on the board.	1	2	3	4	5
4. Taking a maths test.	1	2	3	4	5
5. Being given maths homework with lots of difficult questions that you have to hand in the next day.	1	2	3	4	5
6. Listening to the teacher talk for a long time in maths.	1	2	3	4	5
7. Listening to another child in your class explain a maths problem.	1	2	3	4	5
8. Finding out you are going to have a surprise maths quiz when you start your maths lesson.	1	2	3	4	5
9. Starting a new topic in maths.	1	2	3	4	5
